# Transcriptomic Screening and Functional Characterization of Immune-Related Genes in *Tetranychus urticae* Responding to *Isaria cateniannulata* Infection

**DOI:** 10.3390/insects17080855

**Published:** 2026-08-17

**Authors:** Xue Yang, Na Ding, Lingdi Gu, Can Liu, Zonghua Li, Xin Lu, Yangyang Shi, Weicheng Yang, Ziye Meng, Qingfu Chen, Xiaona Zhang

**Affiliations:** 1 Research Center of Buckwheat Industry Technology, College of Life Science, Guizhou Normal University, Guiyang 550025, China; 242100100450@gznu.edu.cn (X.Y.); 252100100449@gznu.edu.cn (N.D.);; 2 School of International Education, Guizhou Normal University, Guiyang 550025, China; 3 College of Life Science, Guizhou Normal University, Guiyang 550025, China

**Keywords:** *Tetranychus urticae*, *Isaria cateniannulata*, transcriptome, RNA interference, *TuAPOD*, *TuPLA2*

## Abstract

Clarifying the immune response mechanisms of *Tetranychus urticae* against *Isaria cateniannulata* is critical for developing sustainable pest control strategies. In this study, we employed transcriptome sequencing to identify immune-related genes responsive to *Isaria cateniannulata* infection, followed by integrative analysis using qRT-PCR, bioinformatics analysis and RNA interference (RNAi) to systematically study the expression patterns and functions of key candidate genes. The results indicated that a total of 342 genes were significantly differentially expressed in *Tetranychus urticae* following *Isaria cateniannulata* infection, including 322 up-regulated genes and 20 down-regulated genes. These differentially expressed genes were mainly enriched in immune-related pathways, including lysosomes, autophagy, phagosomes, apoptosis and NOD-like receptor signaling. *Isaria cateniannulata* infection resulted in different expression levels of candidate genes across various developmental stages of *Tetranychus urticae*. Among these, *TuAPOD* and *TuPLA2* were highly expressed across all developmental stages, and their encoded proteins—comprising α-helices, extended strands, and random coils—shared high sequence similarity with homologs from other arthropods. RNAi-mediated silencing of these two genes achieved over 75% efficiency at 36 h. Combining RNAi with *Isaria cateniannulata* treatment significantly increased mortality and suppressed oviposition, with the highest 72 h mortality rate observed in the *TuPLA2* + H13 treatment group (98.33%). This study provides preliminary insight into the molecular mechanism underlying *Tetranychus urticae*’s response to *Isaria cateniannulata* infection, clarifies the key role of lipid metabolism-related genes in antifungal immunity, and further confirms that combining RNA interference with *Isaria cateniannulata* treatment exerts a significant synergistic effect. These findings offer new insights and a theoretical basis for developing environmentally friendly strategies for controlling *Tetranychus urticae*.

## Simple Summary

*Tetranychus urticae* is a destructive agricultural pest, and *Isaria cateniannulata* is an important entomopathogenic fungus used for its biological control. To better understand the immune response of *Tetranychus urticae* to fungal infection, we identified immune-related genes using transcriptome sequencing and investigated the functions of key genes through qRT-PCR, bioinformatics analysis, and RNA interference (RNAi). A total of 342 differentially expressed genes were identified after fungal infection, with many involved in immune-related pathways. Silencing *TuAPOD* and *TuPLA2* significantly increased mite mortality and reduced oviposition when combined with *Isaria cateniannulata* treatment. These results improve our understanding of the antifungal immune response in *Tetranychus urticae* and provide a theoretical basis for developing more effective and environmentally friendly biological control strategies.

## 1. Introduction

*Tetranychus urticae* Koch (*T. urticae)*, also known as the two-spotted spider mite, belongs to the phylum Arthropoda, class Arachnida, order Acariformes, family Tetranychidae and genus *Tetranychus* [1]. It is a globally significant polyphagous agricultural pest mite [2]. This species feeds on plant cell contents through piercing-sucking mouthparts, causing chlorotic spots on leaves and reducing photosynthetic efficiency, which ultimately leads to substantial declines in crop yield and quality [3,4]. In addition, its web-spinning behavior can disrupt leaf gas exchange and transpiration, further exacerbating physiological damage to the host plants [5]. Due to its short life cycle, high reproductive capacity, and strong environmental adaptability, *T. urticae* infests more than 1400 plant species, including buckwheat, soybean, maize and a wide range of horticultural crops [6,7,8,9]. In the United States, *T. urticae* has caused significant economic losses in strawberry production. Field experiments conducted in north-central Florida demonstrated that severe infestations of *T. urticae* significantly reduced marketable strawberry yields, highlighting its potential threat to high-value fruit crops [10].

At present, chemical acaricides remain the main means of controlling *T. urticae*. However, long-term and high-intensity application has led to varying degrees of resistance to multiple agents in this pest while also causing environmental pollution, non-target biological hazards, and risks to agricultural product safety [11]. Consequently, developing efficient, safe, and sustainable green control technologies has become a major research focus. Among these, entomopathogenic fungi have attracted considerable attention because they can directly infect and kill arthropods through contact with the cuticle [12].

*Isaria cateniannulata* (*I. cateniannulata*), belonging to Hypocreales of Sordariomycetes, is an entomopathogenic fungus with high spore production, strong adaptability and environmental friendliness [13]. It has been shown to exert a good control effect on *T. urticae* under laboratory and field conditions [12,14,15]. Infection occurs when spores attach to the host surface, germinate to form germ tubes, and differentiate into penetration structures that invade the host body, ultimately causing death [14,16]. However, in the early stage of infection, fungal hyphae can already be observed on and within the mite, while the host may still maintain a certain level of activity, indicating that physiological regulation has not yet been completely lost.

During infection, the first line of defense in *T. urticae* consists of physical barriers, including the cuticle and digestive tract [17]. As the infection progresses, the host activates cellular and humoral immune responses, including hemocyte-mediated phagocytosis and encapsulation; production of immune effector molecules [18]; detoxification enzymes, such as cytochrome P450, glutathione S-transferase, and carboxylesterase; and antioxidant enzymes, including superoxide dismutase, catalase, and peroxidase [19,20,21,22,23,24,25]. Although previous studies have provided insights into the pathogenicity of entomopathogenic fungi and the host immune responses, studies on *Beauveria bassiana* have largely focused on its potential as a biological control agent against *T. urticae*, particularly its pathogenic mechanisms and biocontrol efficacy [26,27]. In contrast, systematic research on the molecular mechanism underlying the host immune response to *I. cateniannulata* infection remains limited, especially regarding the identification and functional characterization of key immune-related genes.

Therefore, in this study, *T. urticae* infected with *I. cateniannulata* was used as the research subject. Transcriptomic analysis was employed to identify host immune-related candidate genes, and their expression patterns were examined using qRT-PCR. Selected candidate genes were further cloned and analyzed through bioinformatics, and their functions were validated using RNA interference (RNAi). The results are expected to elucidate the molecular mechanism underlying *T. urticae*’s response to entomopathogenic fungal infection and provide a theoretical basis for the efficient application of entomopathogenic fungi in environmentally sustainable mite management.

## 2. Materials and Methods

### 2.1. Test Mites

*T. urticae* was derived from the laboratory population of the Buckwheat Engineering Technology Research Center of Guizhou Normal University and was reared in an artificial climate box for a long time. Tartary buckwheat was used as the host plant, and the feeding conditions were a temperature of 25 ± 1 °C, a relative humidity of 80 ± 5%, and a light cycle of L:D = 16:8.

To obtain developmentally synchronized experimental materials, 30 adult female mites were placed on fresh Tartary buckwheat leaves (approximately 4 cm × 4 cm) for oviposition. After 24 h, the adult females were removed, leaving only the eggs to continue development. Under these conditions, the eggs were cultured for 10–12 days, and newly emerged adult females were subsequently collected for use in further experiments.

### 2.2. Test Strains

*I. cateniannulata* strain 2019BY-4 (laboratory number Ring 13) was collected from the soil in Guizhou and preserved in the Buckwheat Engineering Technology Research Center of Guizhou Normal University. The strain was inoculated into liquid PDA medium and incubated at 25 °C and 150 r/min for 5 days. After incubation, the resulting conidial suspension was obtained by filtering through double-layered filter paper [28]. The number of conidia was determined using a hemocytometer (Shanghai Medical Instruments Co., Ltd., Shanghai, China), and the conidial concentration was subsequently adjusted to 2 × 10^7^ conidia/mL for further experiments.

### 2.3. Treatment at Different Time Points

Fresh Tartary buckwheat leaves (4 cm × 4 cm) were placed in a laboratory-constructed hydroponic setup, and 50 adult female mites were introduced onto each leaf. The mites were uniformly sprayed with 0.2 mL of the conidial suspension (2 × 10^7^ conidia/mL), while the control group was sprayed with an equal volume of sterile water [29]. Samples were collected at 4, 12, 24, 36 and 48 h post-treatment, with 200 adult females collected per time point. The samples were rapidly frozen in liquid nitrogen and stored at −80 °C. Three biological replicates were prepared for each treatment.

### 2.4. Treatment of Different Developmental Stages

Eggs, larvae, nymphs and adults of *T. urticae* were separately collected and placed on fresh Tartary buckwheat leaves (50 individuals per leaf). Each developmental stage was treated with the same concentration of conidial suspension, while the control group was sprayed with sterile water. After 36 h of treatment, 200 individuals per developmental stage were collected, rapidly frozen in liquid nitrogen, and stored at −80 °C. Three biological replicates were conducted for each treatment.

### 2.5. RNA Extraction and Quality Control

Total RNA was extracted using TRIzol reagent (Invitrogen, Carlsbad, CA, USA) according to the manufacturer’s instructions (Life Technologies, Carlsbad, CA, USA). RNA concentration and purity were determined using an ultramicro spectrophotometer (Shanghai Yuanxi Instrument Co., Ltd., Model: B-500, Shanghai, China), and the integrity was assessed using 1.2% agarose gel electrophoresis. Only RNA samples with OD260/280 between 1.8 and 2.2 and with complete and clear bands were selected for subsequent analysis.

### 2.6. cDNA Library Construction and Sequencing

Qualified RNA samples were used to enrich mRNA with Oligo (dT) magnetic beads, followed by fragmentation. Subsequently, the first and second strands of cDNA were synthesized with random primers. After purification, the cDNA underwent end repair, A-tailing, and adapter ligation, with the final library constructed via PCR amplification. Library quality was assessed using a Qubit 2.0 Fluorometer (Thermo Fisher Scientific, Waltham, MA, USA) and an Agilent 2100 Bioanalyzer (Agilent Technologies, Santa Clara, CA, USA). Finally, qualified libraries were subjected to high-throughput sequencing on the Illumina HiSeq platform.

### 2.7. Identification and Functional Analysis of Differentially Expressed Genes (DEGs)

Gene expression levels were estimated based on sequencing data, and differential expression analysis was performed using DESeq2 software (version 1.10.1) [30]. The selection criteria for DEGs were set at |log_2_ fold change| ≥ 1 and a false discovery rate (FDR) ≤ 0.01. Differentially expressed genes (DEGs) were functionally classified using the Gene Ontology (GO) database, and significant enrichment analysis was performed using R software (version 3.0.8) [31]. Additionally, DEGs were mapped to the KEGG database for metabolic and signaling pathway enrichment analysis to elucidate their potential biological functions.

### 2.8. Quantitative Real-Time PCR (qRT-PCR) Validation

To validate the gene expression data, qRT-PCR was conducted using the TB Green^®^ Premix Ex Taq^TM^ II kit (Takara Bio Inc., Kusatsu, Shiga, Japan), with α-tubulin employed as the internal reference gene. The reaction system was 25 μL, including specific primers (Table S3), a cDNA template, and nuclease-free water. The amplification procedure was as follows: pre-denaturation at 95 °C for 30 s, followed by 40 cycles of denaturation at 95 °C for 5 s and annealing at 60 °C for 30 s. Each sample was analyzed with three biological and three technical replicates. The relative expression levels of the target genes were calculated using the 2 ^^ −ΔΔCt^ method.

### 2.9. Bioinformatics Analysis

Using the gene-specific primers listed in Table S4, PCR amplification was performed with the cDNA template obtained by reverse transcription. The amplified products were separated and detected using 1% agarose gel electrophoresis. Sequence assembly was conducted using DNAMAN software (version 9.0), while homology searches were performed using NCBI BLAST. The open reading frame (ORF) was predicted using ORF Finder. Conserved domains were analyzed using the Pfam database, and subcellular localization was predicted using WOLF PSORT. The phylogenetic tree was constructed using MEGA software (version 7.0) (neighbor-joining method, bootstrap = 1000). The physicochemical properties and structural characteristics of the proteins were analyzed using ExPASy (web.expasy.org/protparam), TMHMM (version 2.0) and SignalP (version 6.0).

### 2.10. dsRNA Synthesis and RNA Interference (RNAi)

Specific primers (300–600 bp) were designed according to the target gene sequences, with the T7 promoter sequence added to the 5’ end. The purified PCR products served as templates to synthesize dsRNA using an in vitro transcription kit. The integrity of the synthesized dsRNA was assessed by agarose gel electrophoresis, and the concentration was quantified before storage at −80 °C. RNAi treatment was carried out using the leaf-feeding method. The dsRNA solution (200 ng/μL) was applied uniformly onto the surface of Tartary buckwheat leaves. Once the solution was absorbed, adult females that had been starved for 24 h were transferred to the treated leaves. The mites were maintained at 25 ± 1 °C, and the leaves were replaced every 2 days.

### 2.11. Silencing Efficiency Detection

Samples were collected at 24, 36 and 48 h post-RNAi treatment. Total RNA was extracted and reverse-transcribed into cDNA. The expression levels of the target genes were quantified by qRT-PCR to evaluate the interference efficiency.

### 2.12. Bioassay

After identifying the optimal silencing time point, the *T. urticae* individuals were treated with a conidial suspension (2 × 10/mL) using the spray method. After treatment, the mites were transferred to fresh leaves and maintained at 25 ± 1 °C. Mortality, escape rate and egg production were recorded every 12 h post-treatment for a continuous observation period of 72 h.

### 2.13. Data Analysis

All experimental data were presented as the mean ± standard error (mean ± SE). Statistical analyses were performed using SPSS (version 27.0). Differences among treatments were evaluated by one-way ANOVA, followed by Tukey’s multiple comparisons test (*p* < 0.05). The charts were drawn using Origin software (version 2022). Differential expression analysis of transcriptome sequencing data was conducted using the DESeq2 package, with |log2 fold change| ≥ 1 and FDR < 0.01 set as the thresholds for significant differential expression.

## 3. Results and Analysis

### 3.1. Correlation Analysis of Differentially Expressed Genes

PCA showed that PC1 and PC2 explained 39.73% and 28.41% of the total variance, respectively (cumulatively 68.14%). In the PCA plot (Figure S1a), the control group (CK) and the treatment group (ETG) were significantly separated along the PC1 axis, indicating that *I. cateniannulata* infection significantly changed the transcriptional expression profile of *T. urticae*. Replicates within the CK group clustered closely, showing high reproducibility, while the ETG group showed some dispersion, potentially due to varying degrees of infection or individual biological differences. Correlation analysis (Figure S1b) showed that intra-group correlation coefficients were all >0.95, while the inter-group correlation remained low. Hierarchical clustering also clearly distinguished CK from ETG. In summary, these results confirm high data reliability, making the data suitable for subsequent differential expression analysis.

### 3.2. Differential Gene Expression Analysis

**Figure 1:**
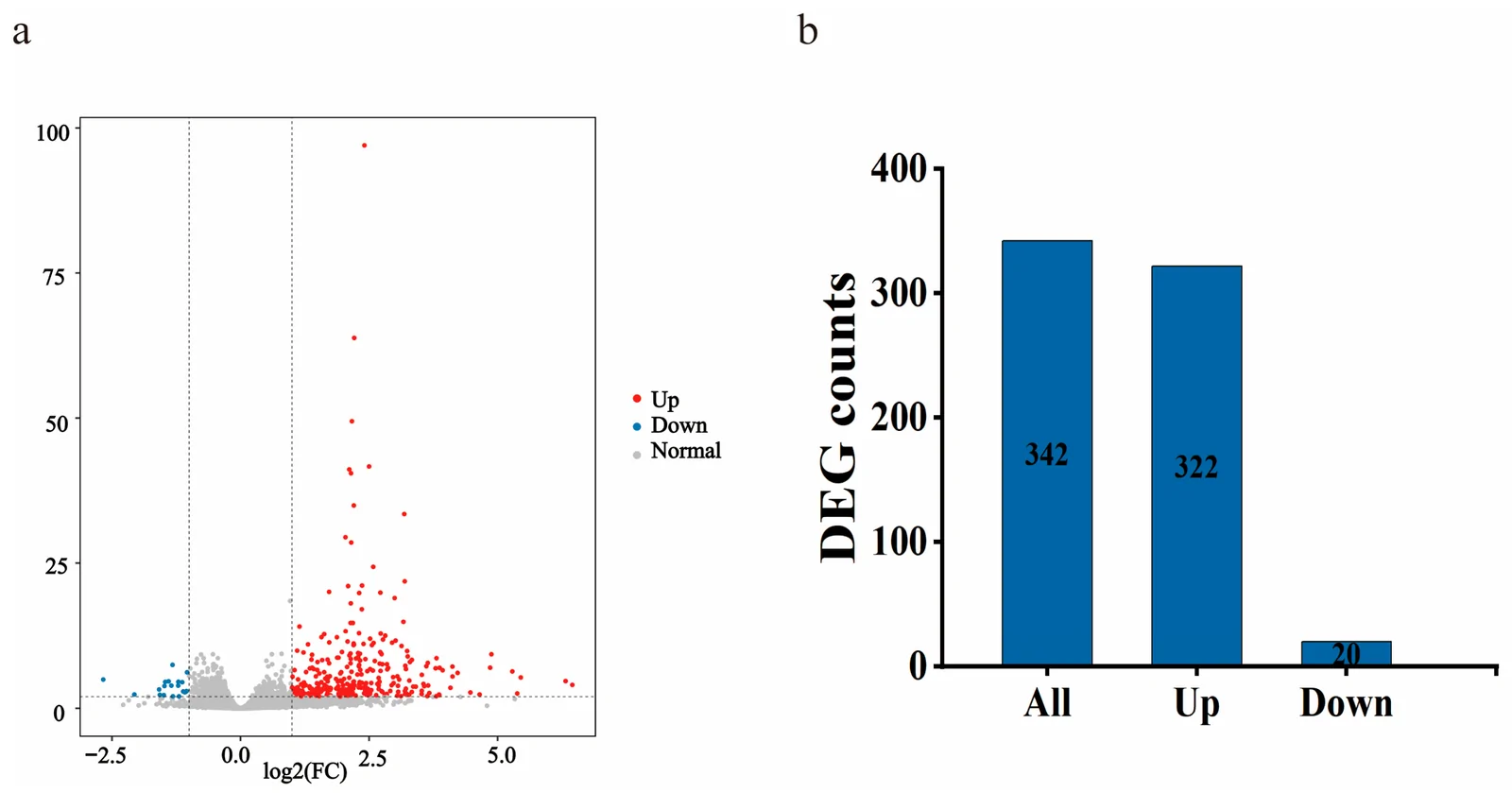
Volcano plot and quantitative distribution of differentially expressed genes. The volcano plot on the left (**a**) provides an overview of the differential gene expression profile, with up-regulated genes highlighted in red, down-regulated genes in blue, and non-DEGs in gray. The bar chart on the right (**b**) presents the statistical distribution of up- and down-regulated DEGs.

The impact of *I. cateniannulata* infection on gene expression in *T. urticae* was visualized in Figure 1. Compared with the control group, a total of 342 differentially expressed genes (DEGs) were identified. Among these, 322 genes were significantly up-regulated, while 20 genes were significantly down-regulated (Figure 1), indicating that *I. cateniannulata* infection activated a large number of genes in *T. urticae*.

### 3.3. GO and KEGG Functional Enrichment Analysis of DEGs

**Figure 2:**
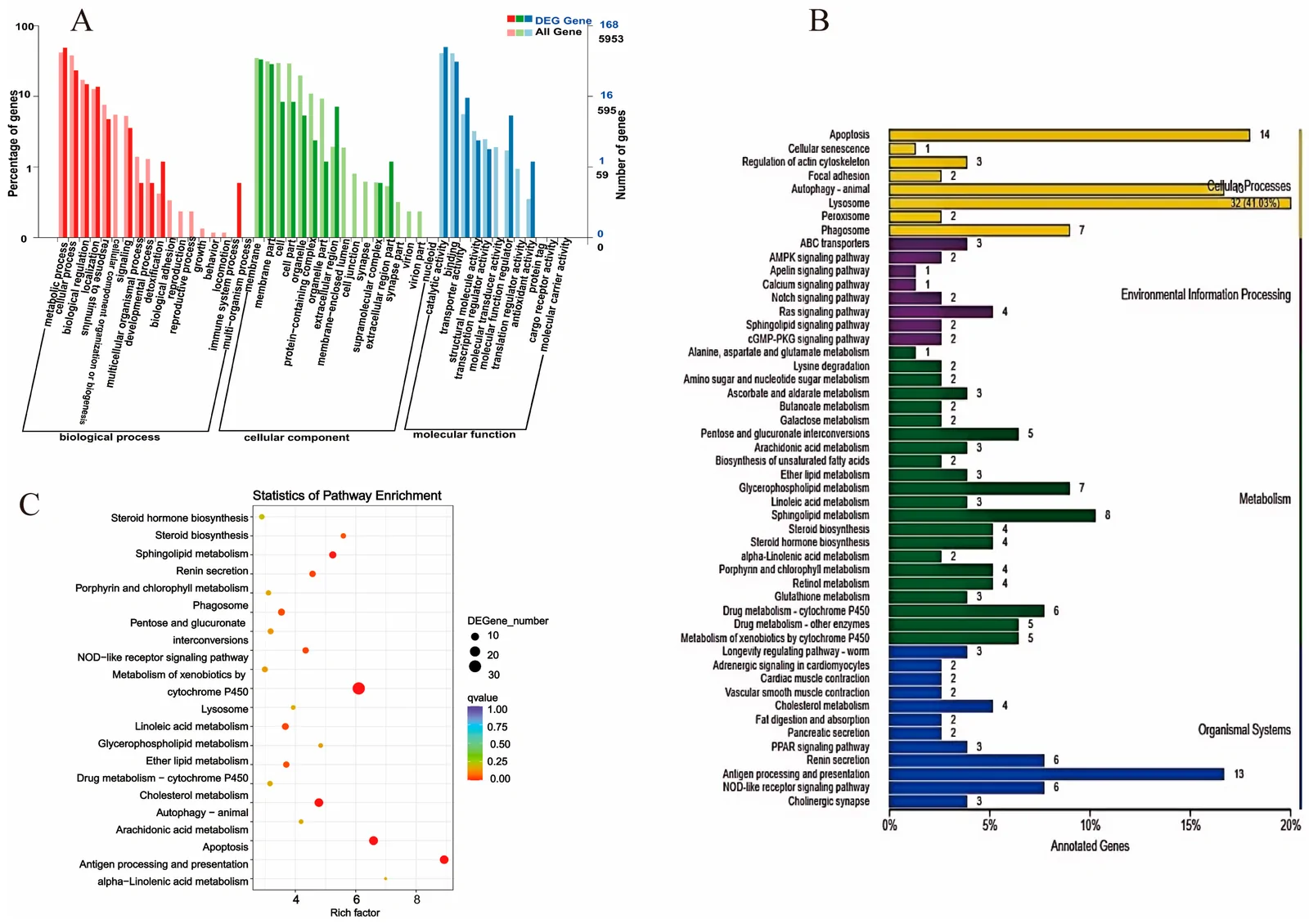
Functional annotation and enrichment analyses of differentially expressed genes (DEGs) identified following *Isaria cateniannulata* infection in *Tetranychus urticae*. (**A**) Gene Ontology (GO) classification of DEGs. GO terms are categorized into three functional groups: biological process (BP), cellular component (CC), and molecular function (MF). Dark-colored bars represent DEGs, whereas light-colored bars represent all annotated genes. The left y-axis indicates the percentage of genes, and the right y-axis indicates the number of DEGs. (**B**) Kyoto Encyclopedia of Genes and Genomes (KEGG) pathway classification of DEGs. The x-axis represents the percentage of annotated genes assigned to each pathway, and the numbers beside the bars indicate the number of DEGs enriched in each pathway. Pathways are grouped into the categories of cellular processes, environmental information processing, metabolism, and organismal systems. (**C**) KEGG pathway enrichment analysis of DEGs. The x-axis represents the Rich factor, calculated as the ratio of the number of DEGs enriched in a pathway to the total number of genes annotated in that pathway. The size of each circle indicates the number of enriched DEGs (DEGene_number), and the color represents the adjusted *p*-value (*q*-value). Larger circles indicate more enriched genes, and lower *q*-values indicate higher enrichment significance. Abbreviations: GO, Gene Ontology; KEGG, Kyoto Encyclopedia of Genes and Genomes; DEG, differentially expressed gene; BP, biological process; CC, cellular component; MF, molecular function.

Functional classification of the DEGs was performed across three main Gene Ontology (GO) categories: biological processes (16 terms), cell composition (10 terms), and molecular functions (8 terms) (Figure 2A). In the biological process (BP) category, DEGs were mainly enriched in important biological processes such as metabolic processes, cellular processes, biological regulation and localization, which indicates that *I. cateniannulata* infection can activate various metabolic activities and cellular regulatory processes in *T. urticae* to cope with fungal stress. In the cell composition (CC) category, DEGs were mainly associated with membrane-related structures, membrane parts, organelles, and extracellular regions. This indicates that pathogen infection may cause changes in cell membrane structure and organelles, thereby affecting cell signal transduction and material exchange. In the molecular function (MF) category, enrichment was observed in catalytic activity, binding, and transporter activity. These functions are closely linked to enzymatic reactions, molecular recognition and material transport, indicating that the host regulates these interactions to participate in immune defense and metabolic regulation.

Further analysis of the biological pathways using the KEGG database (Figure 2B) showed that 78 DEGs were enriched in 83 pathways, categorized into four primary classes: cellular processes, environmental information processing, metabolism and organismal systems. Among them, the metabolism-related category contained the highest number of DEGs, highlighting a significant metabolic shift in *T. urticae* following infection. An analysis of the top 20 pathways with the lowest q-values (Figure 2C) identified eight pathways closely related to host immune defense and immune-related metabolism. These pathways mainly include lysosomes, antigen processing and presentation, autophagy–animal, apoptosis, phagosomes, the NOD-like receptor signaling pathway, sphingolipid metabolism, and glycerophospholipid metabolism.

Based on a selection threshold of more than a fourfold expression difference and relevance to immune function, a total of eight candidate genes were prioritized for further study: lipid metabolism-related genes (*TuAPOD*, *TuLip*, and *TuPLA2*), detoxification and xenobiotic metabolism genes (*TuCYP1* and *TuCYP2*), stress and transcriptional regulation genes (*TuTrp* and *TuMaf*), and binding and transport-related genes (*TuRbp*) (Table S1).

### 3.4. Expression Characteristics of Candidate Genes at Different Time Points

**Figure 3:**
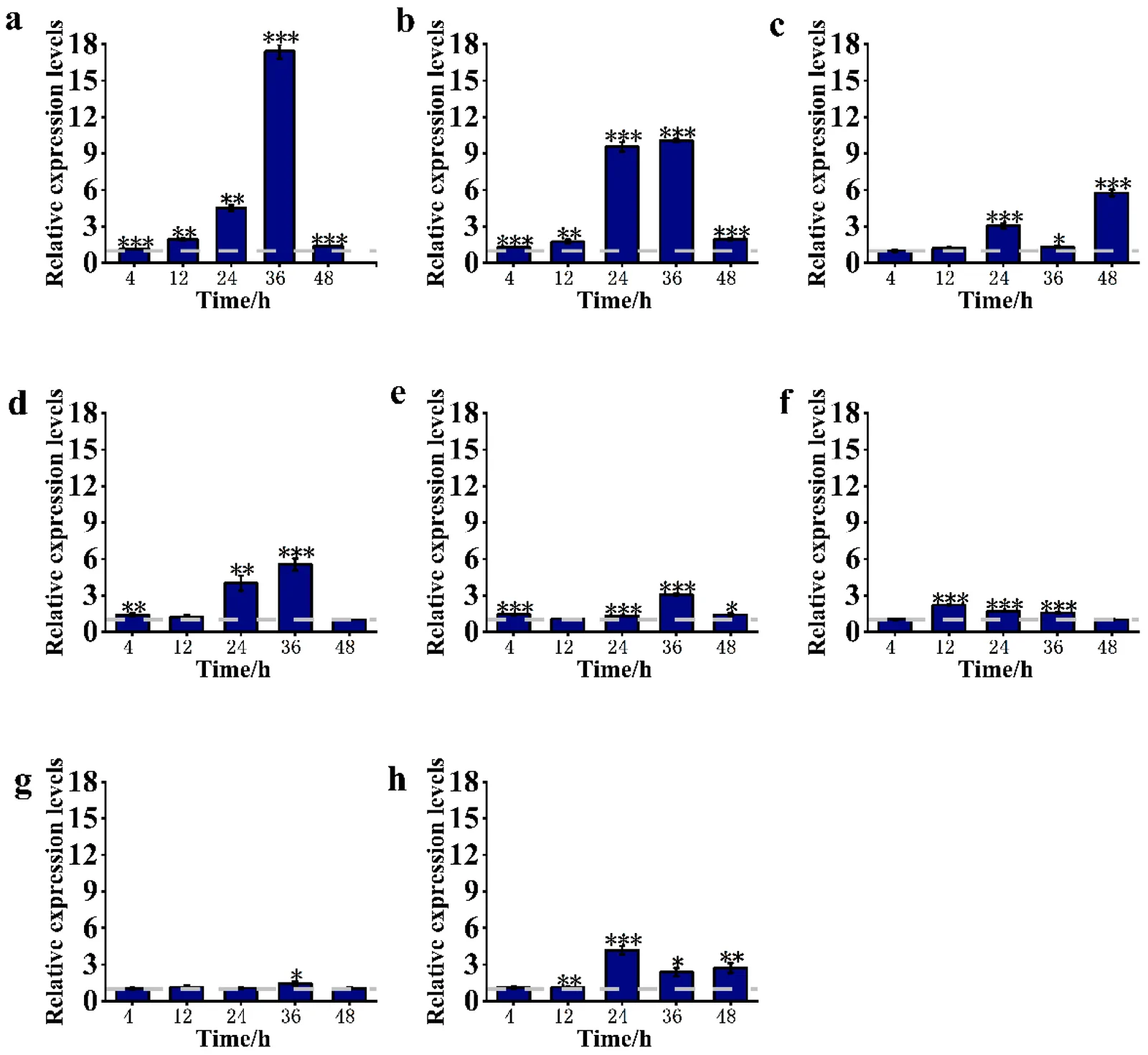
Quantitative analysis of immune-related genes in *Tetranychus urticae* treated with *Isaria cateniannulata* at different time points. (**a**) Expression of *TuAPOD*. (**b**) Expression of *TuPLA2*. (**c**) Expression of *TuLip*. (**d**) Expression of *TuCYP1*. (**e**) Expression of *TuCYP2*. (**f**) Expression of *TuMaf*. (**g**) Expression of *TuTrp*. (**h**) Expression of *TuRbp*. Values are mean ± SE. * *p* < 0.05, ** *p* < 0.01, *** *p* < 0.001 (independent-samples *t*-test). The dashed line indicates the relative expression level of 1 in the control group.

The expression levels of eight candidate genes in adult *T. urticae* initially increased and subsequently decreased after infection with *I. cateniannulata.* The expression levels of *TuAPOD* (Figure 3a), *TuPLA2* (Figure 3b), *TuCYP2*, *TuTrp* and *TuRbp* reached their highest levels at 36 h, showing 17.44-, 10.08-, 3.08-, 1.46- and 5.54-fold increases relative to the control group, respectively (Figure 3e,g,h). In contrast, *TuLip* and *TuCYP1* exhibited the highest expression at 24 h, with expression levels 3.07- and 4.20- fold higher than those of the control group, respectively (Figure 3c,d). The expression level of *TuMaf* peaked at 12 h, reaching 2.23-fold that of the control group (Figure 3f).

### 3.5. Expression Characteristics of Candidate Genes at Different Developmental Stages

**Figure 4:**
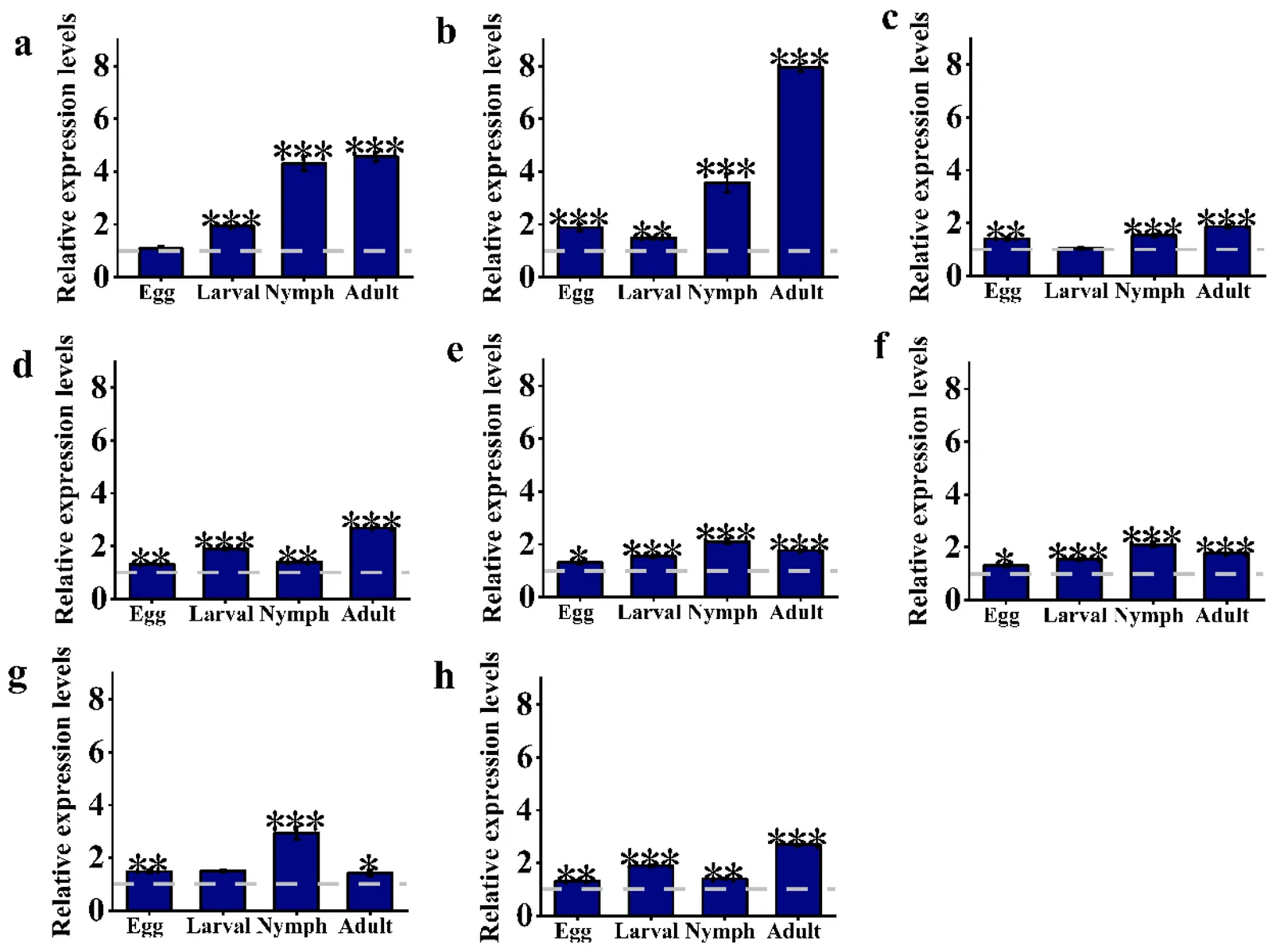
Quantitative analysis of immune-related genes in *Tetranychus urticae* at different developmental stages treated with *Isaria cateniannulata.* (**a**) Expression of *TuAPOD*. (**b**) Expression of *TuPLA2*. (**c**) Expression of *TuLip*. (**d**) Expression of *TuCYP1. (***e**) Expression of *TuCYP2. (***f**) Expression of *TuMaf*. (**g**) Expression of *TuTrp*. (**h**) Expression of *TuRbp.* Values are mean ± SE. * *p* < 0.05, ** *p* < 0.01, *** *p* < 0.001 (independent-samples *t*-test). The dashed line indicates the relative expression level of 1 in the control group.

The expression patterns of the eight candidate genes in *T. urticae* varied significantly across developmental stages. The expression level of *TuAPOD* gradually increased during the development of *T. urticae* and reached the highest level in the adult stage, which was 4.57-fold higher than that in the control group (Figure 4a). The expression patterns of *TuPLA2* and *TuLip* showed a ‘V-shaped’ trend, reaching 7.95- and 1.8-fold higher levels than the control group, respectively (Figure 4b,c). In contrast, *TuCYP1* and *TuRbp* showed an initial increase followed by a decline across developmental stages, with peak expression occurring in the adult stage (Figure 4d,h). Similarly, *TuCYP2*, *TuMaf* and *TuTrp* showed an increasing-then-decreasing trend across all developmental stages, with the highest expression levels observed during the nymphal stage, reaching 2.10-, 1.98- and 2.94-fold that of the control group, respectively (Figure 4d–f). Because the mite stage represents a critical period for the control of *T. urticae*, and both *TuAPOD* and *TuPLA2* exhibited the highest expression levels during this stage, these genes were hypothesized to play key roles in immune defense. Therefore, *TuAPOD* and *TuPLA2* were selected for subsequent experiments.

### 3.6. Bioinformatics Analysis of the TuAPOD Gene

**Figure 5:**
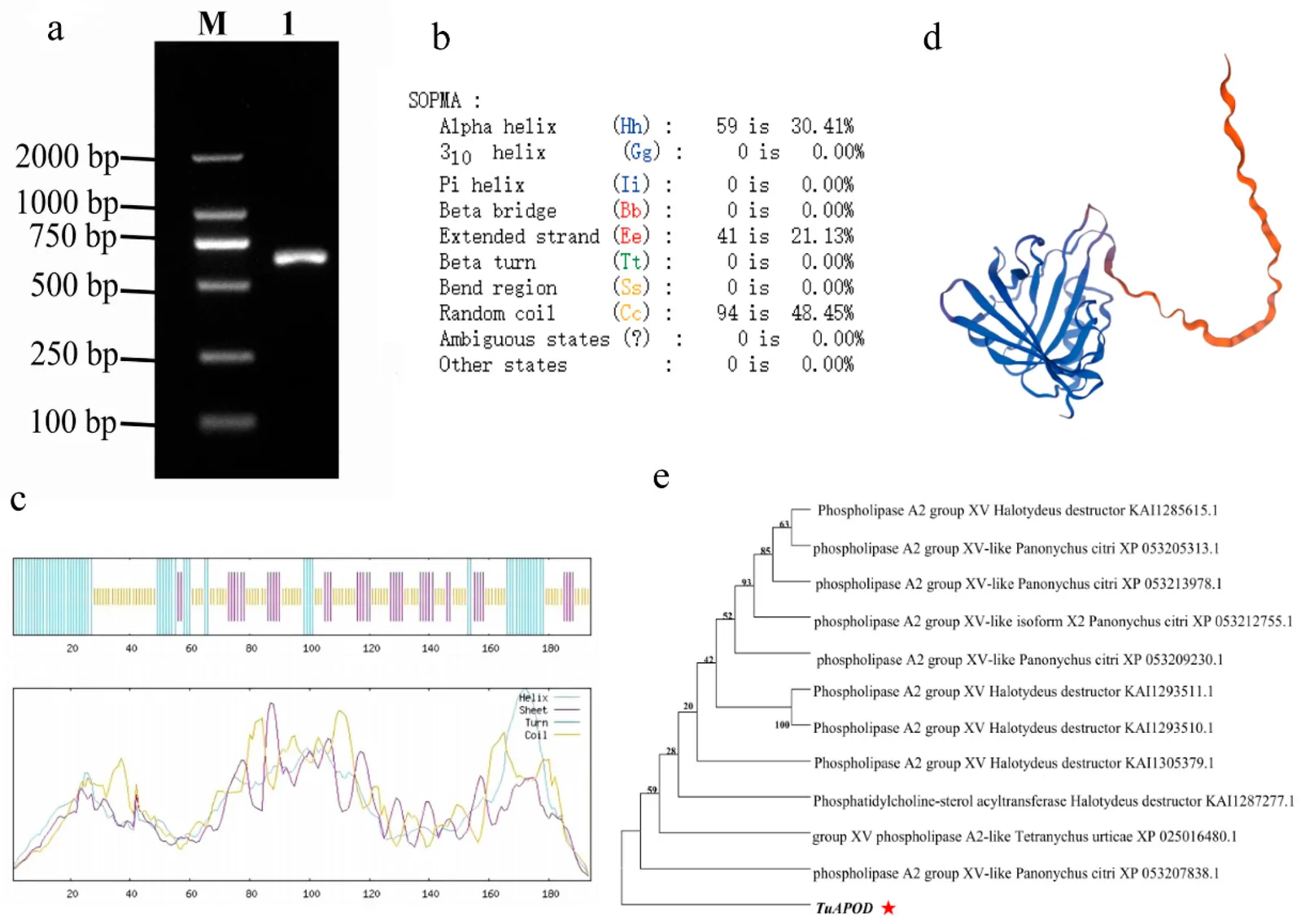
Phylogenetic analysis of the TuAPOD gene. (**a**) PCR amplification of the *TuAPOD* gene. M, DNA marker; Lane 1, PCR product of the *TuAPOD* gene (approximately 600 bp). (**b**) Predicted secondary structure composition of the *TuAPOD* protein, showing the proportions of α-helix, extended strand, random coil, and other structural elements. (**c**) Distribution of the predicted secondary structural elements along the amino acid sequence of the *TuAPOD* protein, blue bars indicate α-helix, purple bars indicate extended strand, and yellow bars indicate *β*-turn. Different-colored lines in the lower profile plot represent helix, sheet, turn, and coil, respectively. (**d**) Predicted three-dimensional (3D) structure model of the *TuAPOD* protein. (**e**) Phylogenetic tree of *TuAPOD* and homologous phospholipase A2 proteins constructed using the neighbor-joining (NJ) method with 1000 bootstrap replicates. Bootstrap support values are shown at the corresponding nodes (only values >50% are displayed). The *TuAPOD* sequence identified in this study is indicated by a red star.

The full-length *TuAPOD* sequence was 585 bp in length (Figure 5a) and encoded a protein of 194 amino acids. The predicted molecular formula of the encoded protein was C_1021_H_1528_N_246_O_288_S_6_, with a molecular weight of approximately 22.05 kDa, and the theoretical isoelectric point (pI) was 4.74. The instability index was 27.11 (<40), and the aliphatic amino acid index was 87.42 (Table 1), indicating that the protein possesses good structural stability and thermal stability.

**Figure 6:**
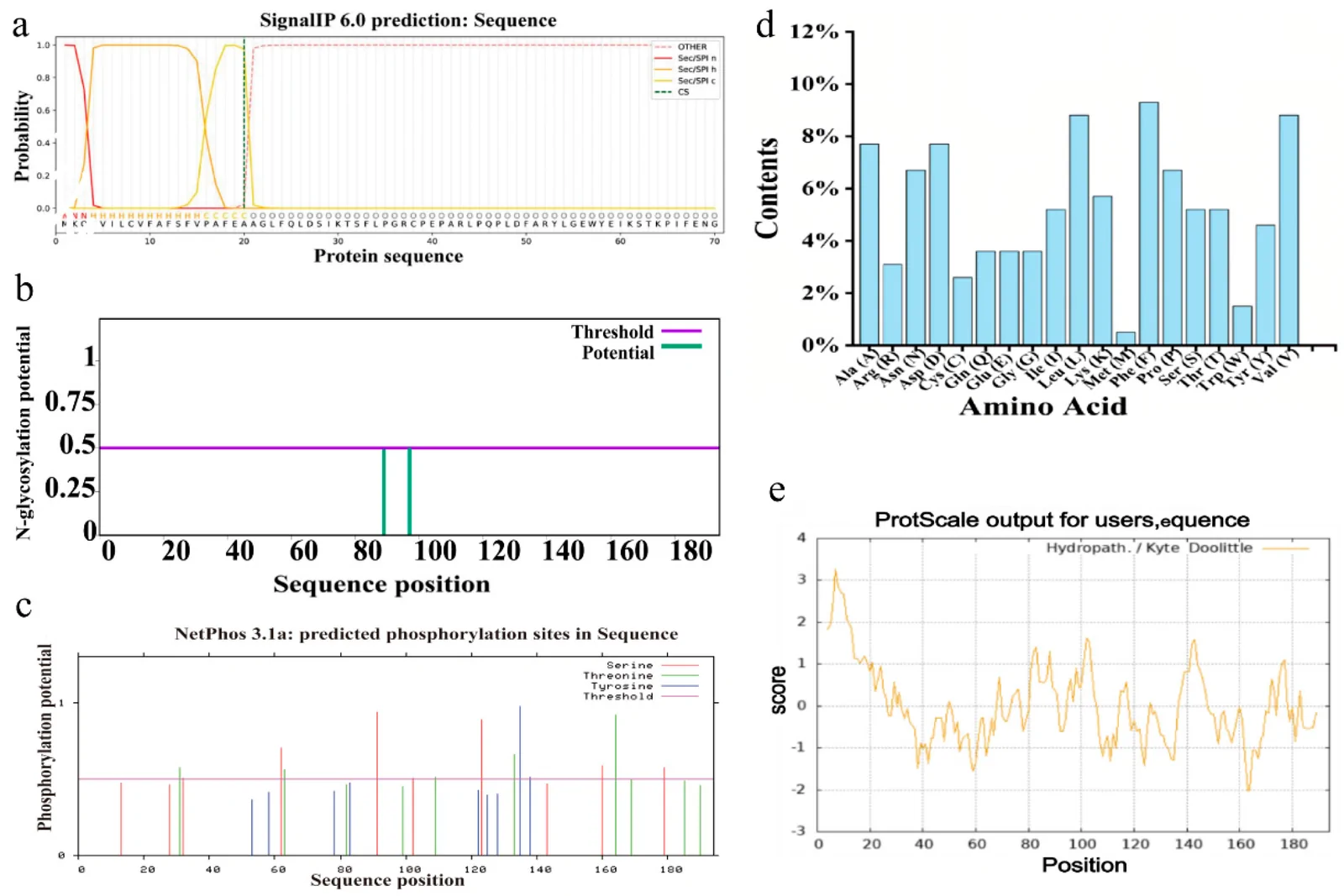
Physicochemical property analysis of the *TuAPOD* gene. (**a**) Signal peptide prediction using SignalP 6.0. The x-axis represents the amino acid positions, and the y-axis indicates the prediction probability. (**b**) Prediction of N-glycosylation sites in the *TuAPOD* protein. The purple horizontal line indicates the prediction threshold, and the green vertical bars represent the predicted N-glycosylation sites. (**c**) Prediction of potential phosphorylation sites using NetPhos 3.1. The red, green, and blue bars represent predicted phosphorylation sites for serine (Ser), threonine (Thr), and tyrosine (Tyr) residues, respectively. The horizontal line indicates the prediction threshold. (**d**) Amino acid composition of the *TuAPOD* protein. The x-axis shows the amino acid residues, and the y-axis indicates the percentage content of each amino acid. (**e**) Hydrophobicity profile of the *TuAPOD* protein based on the Kyte–Doolittle scale, with positive and negative values representing hydrophobic and hydrophilic regions, respectively. Abbreviations: Ser, serine; Thr, threonine; Tyr, tyrosine.

Secondary structure prediction showed that the *TuAPOD* protein consisted mainly of random coils (Cc 48.45%), followed by α-helices (Hh 30.41%), and extended strands (Ee21.13%) (Figure 5b–d). The N-terminal region of the *TuAPOD* protein was predicted to contain a signal peptide sequence with a length of 20 amino acids, with the cleavage site located between the 20th (alanine) and 21st (alanine) residues, indicating that *TuAPOD* is likely to be a secretory protein transported through the classical endoplasmic reticulum–Golgi pathway (Figure 6a). No N-glycosylation sites were predicted for *TuAPOD* (Figure 6b), although it contains 15 phosphorylation sites (Ser, Thr, and Tyr) (Figure 6c). Amino acid composition analysis revealed relatively high proportions of leucine (Leu), glycine (Gly) and aspartic acid (Asp), whereas aromatic amino acids such as tryptophan (Trp) were present at relatively low levels (Figure 6d). This amino acid composition is similar to that of many secretory lipid-binding proteins. Its structure is usually composed of more hydrophobic amino acids, thereby forming a stable lipid-binding pocket for binding and transporting lipid molecules. Further observation showed that the proportion of hydrophilic residues was significantly higher than that of hydrophobic residues, and *TuAPOD* was judged to be a hydrophilic protein (Figure 6e).

The above results indicate that the physicochemical properties of *TuAPOD* are favorable for molecular recognition and ligand interaction in the extracellular environment. In the phylogenetic analysis, *TuAPOD* (marked with a red star) clustered with multiple group XV phospholipase A2-like sequences from Panonychus citri and phosphatidylcholine–sterol acyltransferase (accession number KA11287277.1) from Halotydeus destructor. This branch also contained several group XV PLA2 sequences from other mite species, indicating that *TuAPOD* is closely associated with lipid metabolism-related proteins and belongs to the group XV phospholipase A2 family (Figure 5e).

### 3.7. Bioinformatics Analysis of the TuPLA2 Gene

**Figure 7:**
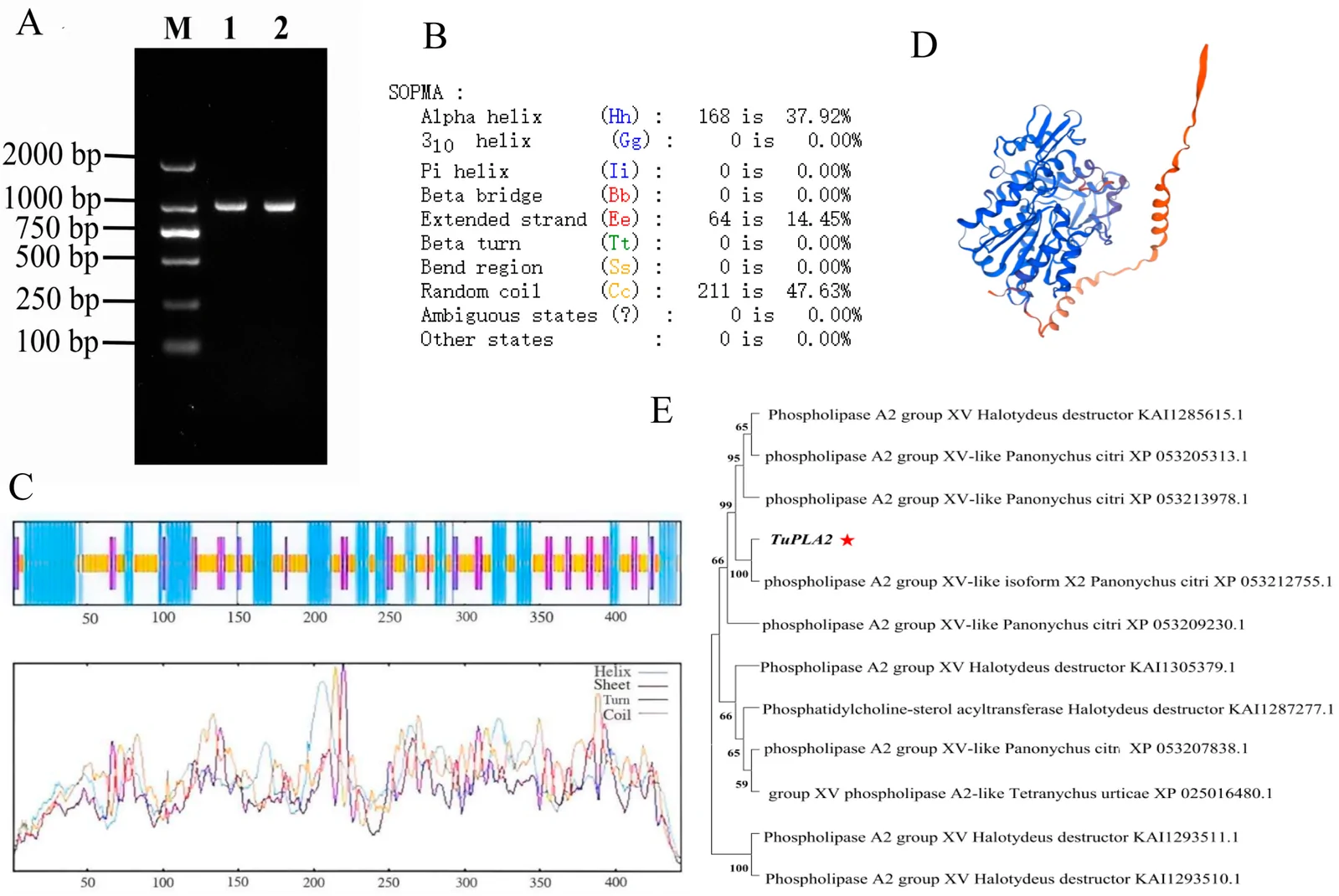
Phylogenetic analysis of the *TuPLA2* gene. (**A**) PCR amplification of the *TuPLA2* gene. M, DNA marker; Lanes 1–2, PCR products of the *TuPLA2* gene (approximately 900 bp). (**B**) Predicted secondary structure composition of the *TuPLA2* protein, showing the proportions of α-helix, extended strand, random coil, and other structural elements. (**C**) Distribution of predicted secondary structural elements along the amino acid sequence of the *TuPLA2* protein, blue bars indicate α-helix, purple bars indicate extended strand, and yellow bars indicate *β*-turn. Different-colored lines in the lower profile plot represent helix, sheet, turn, and coil, respectively. (**D**) Predicted three-dimensional (3D) structure model of the *TuPLA2* protein. (**E**) Phylogenetic tree of *TuPLA2* and homologous phospholipase A2 proteins constructed using the neighbor-joining (NJ) method with 1000 bootstrap replicates. Bootstrap support values are displayed at the corresponding nodes (only values >50% are shown). The *TuPLA2* sequence identified in this study is marked with a red star.

**Figure 8:**
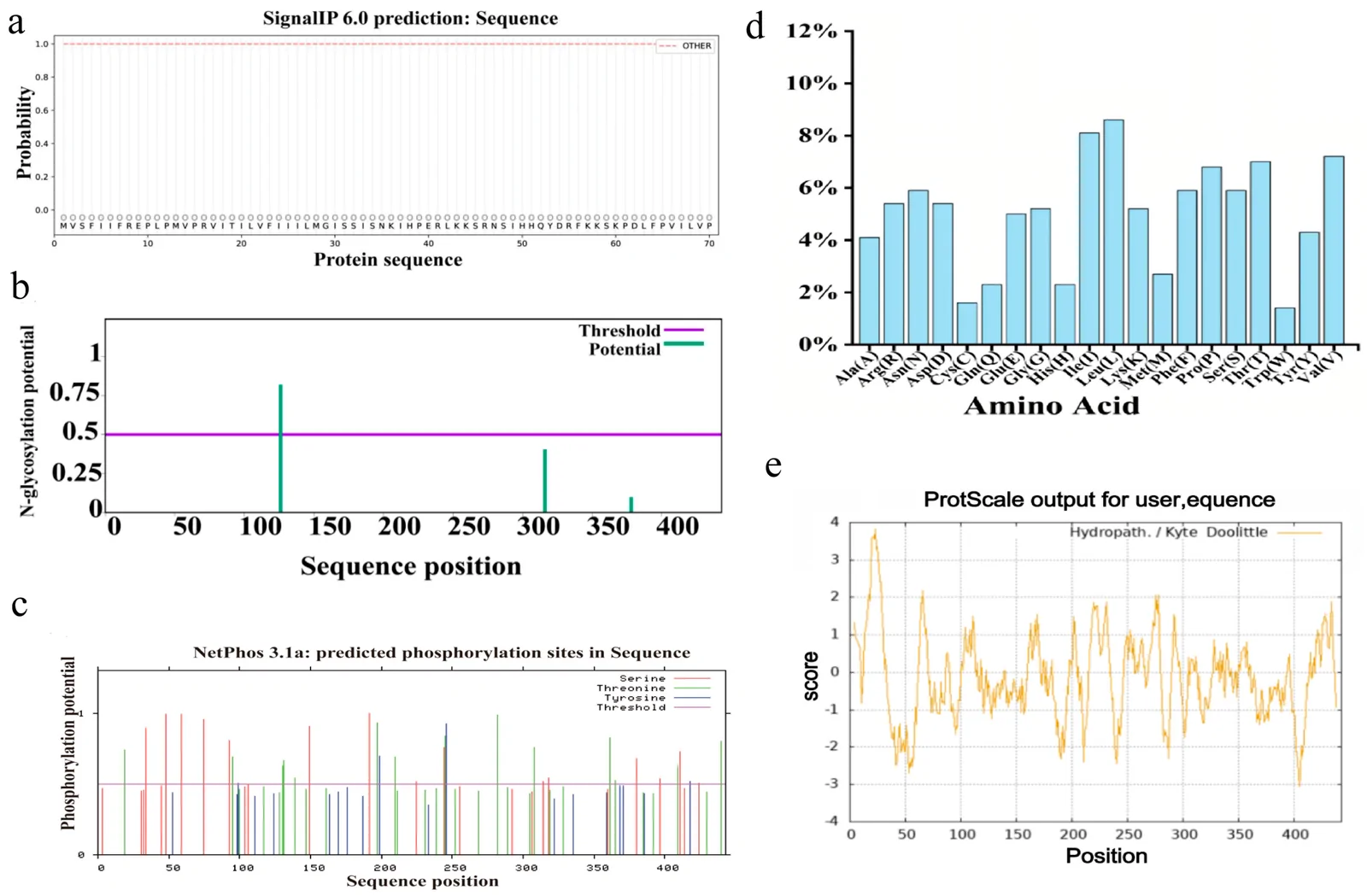
Physicochemical property analysis of the *TuPLA2* gene. (**a**) Signal peptide prediction using SignalP 6.0. The x-axis represents the amino acid positions, and the y-axis indicates the prediction probability. (**b**) Prediction of N-glycosylation sites in the TuPLA2 protein. The purple horizontal line indicates the prediction threshold, and the green vertical bars represent predicted N-glycosylation sites. (**c**) Prediction of potential phosphorylation sites using NetPhos 3.1. The red, green, and blue bars represent predicted phosphorylation sites for serine (Ser), threonine (Thr), and tyrosine (Tyr) residues, respectively. The horizontal line indicates the prediction threshold. (**d**) Amino acid composition of the TuPLA2 protein. The x-axis shows amino acid residues, and the y-axis indicates their relative proportions. (**e**) Hydrophobicity profile of the *TuPLA2* protein based on the Kyte-Doolittle scale. The x-axis represents amino acid positions, and the y-axis represents hydrophobicity scores. Positive and negative values indicate hydrophobic and hydrophilic regions, respectively. Abbreviations: Ser, serine; Thr, threonine; Tyr, tyrosine.

The full-length CDS of the *TuPLA2* gene was 1329bp (Figure 7A), encoding 443 amino acids. The predicted molecular formula is C_2310_H_3572_N_600_O_648_S_19_, with a calculated molecular weight of 50.73 kDa, a theoretical isoelectric point (pl) of 7.61, an instability index of 45.45, and an aliphatic index of 90.16 (Table 1). The secondary structure of the *TuPLA2* protein consists primarily of α-helices (Hh, 37.92%), extended strands (Ee, 14.45%) and random coils (Cc, 47.63%) (Figure 7B,C). The core domain is mainly composed of α-helices and random coils, with the long, laterally extended α-helix potentially mediating molecular recognition or interactions (Figure 7D). No typical signal peptide was predicted, suggesting that *TuPLA2* may not utilize the classical secretory pathway for transmembrane transport (Figure 8a). Post-translational modification analysis identified one N-glycosylation site (Figure 7D) and 34 phosphorylation sites (Ser/Thr/Tyr) (Figure 8c). Amino acid composition analysis showed that leucine (Leu) is the most abundant residue, accounting for 8.60%, followed by hydrophobic residues such as alanine (Ala), valine (Val) and glycine (Gly) (Figure 8d). These residues likely contribute to the formation of the hydrophobic core, stabilizing the protein structure and its enzymatic active center. Furthermore, *TuPLA2* contains a certain proportion of polar amino acids such as serine (Ser) and threonine (Thr). These amino acid residues are often important sites for protein phosphorylation modification and are closely related to signal transduction and enzyme activity regulation. Despite the hydrophobic core, the overall proportion of hydrophilic residues significantly outweighs hydrophobic ones, classifying *TuPLA2* as a hydrophilic protein (Figure 8e).

Phylogenetic analysis revealed that *TuPLA2* (marked with a red star) clustered closely with two group XV phospholipase A2-like sequences from Panonychus citri (XP 053205313.1 and XP 053213978.1), forming a larger branch with the group XV PLA2 of Pityophthorus erythropodus (KA11285615.1). This result clearly indicates that *TuPLA2* belongs to the group XV phospholipase A2 family, sharing the highest homology with *P. citri* (Figure 7E).

### 3.8. Verification of RNAi Silencing Efficiency

**Figure 9:**
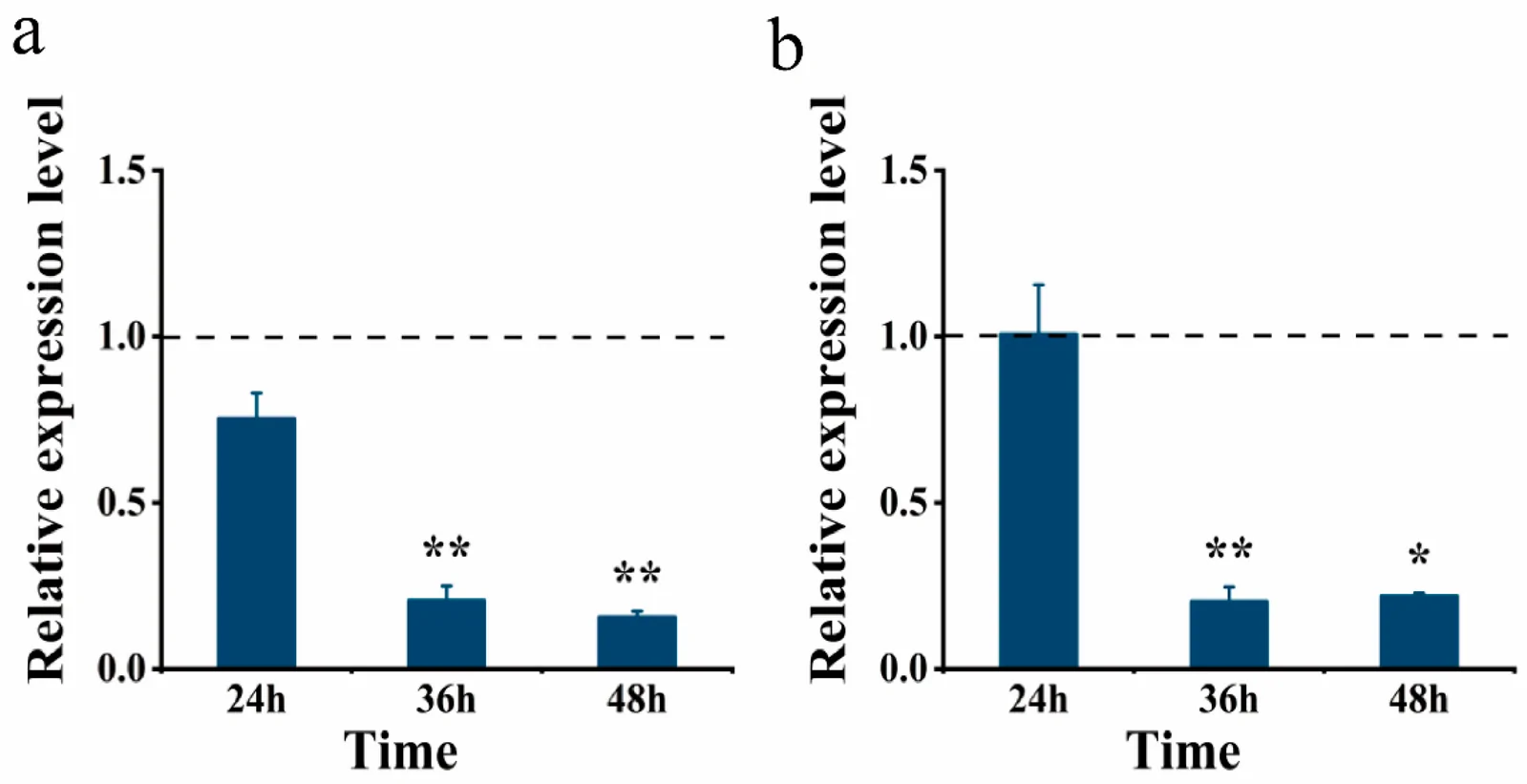
Silencing efficiency of *TuAPOD* and *TuPLA2* genes by RNAi in *T. urticae.* (**a**) Silencing efficiency of the *TuAPOD* gene. (**b**) Silencing efficiency of the *TuPLA2* gene. The dashed line indicates the relative expression level of the control group (set to 1.0). Data are presented as the mean ± SD of three biological replicates. Asterisks indicate significant differences compared with the control group: *p* < 0.05 (*), *p* < 0.01 (**).

In order to verify the regulatory function of *TuAPOD* and *TuPLA2* genes in the immune response of *T. urticae* against *I. cateniannulata*, mites were fed leaves treated with dsRNA. The results showed that the silencing efficiency of *TuAPOD* increased over time, reaching 79.45% at 36 h and 84.4% at 48 h compared to the control (Figure 9a). For *TuPLA2*, no significant change in expression was observed at 24 h, but the silencing efficiency gradually increased, reaching 78.12% at 48 h (Figure 9b). The results fully show that the rational use of dsRNA can effectively inhibit the expression of *TuAPOD* and *TuPLA2* genes in *T. urticae*. Although the gene silencing efficiency peaked at 48 h, the difference compared to 36 h was not statistically significant (*p* > 0.05). Consequently, 36 h was selected as the best interference time point for subsequent experiments.

### 3.9. Analysis of the Biological Effects of RNAi Combined with Isaria Cateniannulata

**Figure 10:**
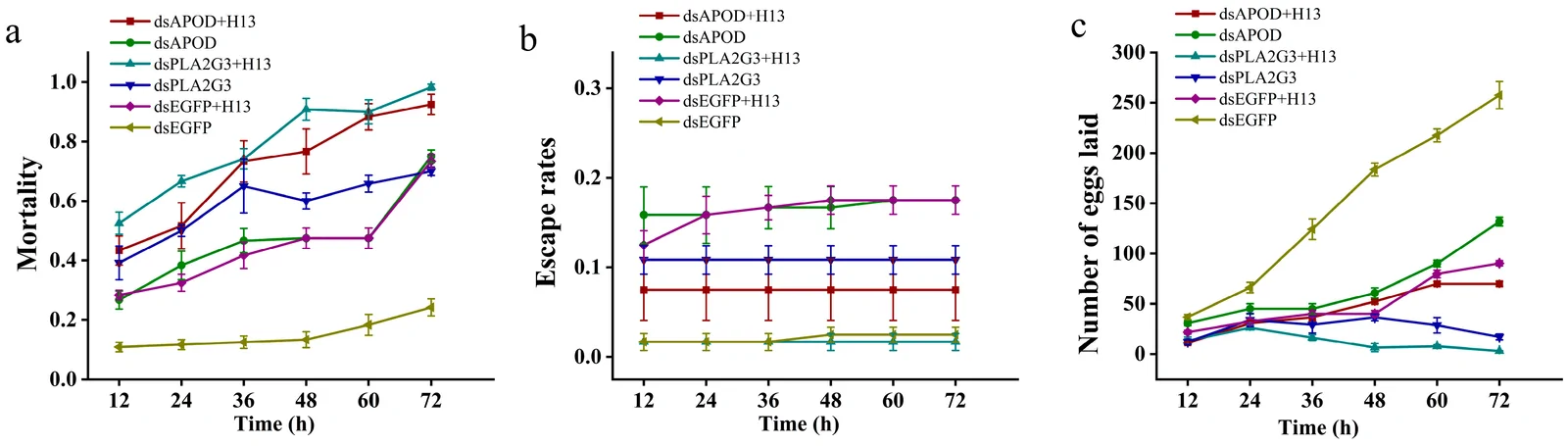
RNAi Silencing of *TuAPOD* and *TuPLA2* Genes. (**a**) Mortality of *T. urticae* following dsRNA treatment and *I. cateniannulata* infection. (**b**) Effects of gene silencing and *I. cateniannulata* infection on the escape rate of *T. urticae*. (**c**) Effects of gene silencing and *I. cateniannulata* infection on oviposition of *T. urticae*. dsAPOD + H13: *I. cateniannulata* inoculation following *TuAPOD* gene silencing; dsAPOD: *TuAPOD* gene silencing; dsPLA2 + H13: *I. cateniannulata* inoculation following *TuPLA2* gene silencing; dsPLA2: *TuPLA2* gene silencing; dsEGFP + H13: negative control dsRNA treatment followed by *I. cateniannulata* inoculation; dsEGFP: negative control dsRNA treatment. dsAPOD and dsPLA2 denote dsRNAs targeting *TuAPOD* and *TuPLA2*, respectively, whereas dsEGFP denotes the negative control dsRNA.

The mortality of adult *T. urticae* increased gradually over time in all treatment groups (Figure 10), peaking at 72 h. At this time point, the mortality rates were 73.33% ± 2.36% for the dsEGFP + *I. cateniannulata* (H13) treatment., 75.00% ± 2.15% for dsAPOD alone, 92.50% ± 3.44% for the dsAPOD + H13 treatment, 70.00% ± 1.36% for dsPLA2 alone, 98.33% ± 0.96% for the dsPLA2 + H13 treatment, and 24.17% ± 2.85% for the dsEGFP control group. These results indicate that dsPLA2 silencing significantly increased the sensitivity of *T. urticae* to *I. cateniannulata* (Figure 10a). Compared with dsPLA2 treatment alone, the combined dsPLA2 + H13 treatment resulted in substantially higher mortality and was approximately 4.06-fold higher than that of the dsEGFP treatment group. Moreover, the combined application of dsRNA and *I. cateniannulata* produced a markedly stronger effect on mortality than either dsRNA treatment alone or the control treatment. Collectively, these findings demonstrate that silencing key immune-related genes enhances the susceptibility of *T. urticae* to fungal pathogens, providing experimental support for the combined application of RNAi and biological control strategies. The effects of combined dsRNA and *I. cateniannulata* treatment on the escape rate of *T. urticae* are shown in Figure 10b. During the whole observation period (12–72 h), no significant changes in escape rate were observed over time in any treatment group, and the overall escape rate remained relatively stable. In the dsAPOD + H13 treatment group, the escape rate consistently remained between 7% and 8%, with only minor fluctuations. The dsPLA2-only treatment group exhibited a slightly higher escape rate, ranging from 10% to 11%, although no significant differences were detected among time points. In contrast, the dsAPOD-only treatment group and the dsEGFP + H13 control group showed relatively higher escape rates, at 15–18%, but these values also remained generally stable throughout the experiment. A comparison among treatments indicated that neither silencing of dsAPOD nor dsPLA2 alone nor their combined application with *I. cateniannulata* induced significant escape behavior in *T. urticae*. These results suggest that under the present experimental conditions, neither target gene silencing nor fungal treatment triggered behavioral avoidance responses in the mites.

The effects of dsRNA combined with *I. cateniannulata* (H13) treatment on the oviposition capacity of *T. urticae* are shown in Figure 10c. The number of eggs laid in all treatment groups was lower than that in the control group. In the dsEGFP, dsAPOD, and dsEGFP + H13 treatment groups, oviposition increased continuously over time, reaching the highest at 72 h, with approximately 91, 132 and 3 eggs, respectively. In contrast, the dsAPOD + H13, dsPLA2, and dsPLA2 + H13 treatment groups showed an initial increase followed by a decrease or stabilization in oviposition over time, with egg numbers at 72 h of approximately 70, 13, 18, and 3, respectively. The results indicate that silencing *TuAPO* or *TuPLA2*, either alone or in combination with fungal treatment, reduced the oviposition capacity of *T. urticae*, and that the combined treatments were more effective than gene silencing alone. Among all treatments, the combination of *TuPLA2* silencing and *I. cateniannulata* infection produced the strongest inhibitory effect on oviposition.

## 4. Discussion and Conclusions

RNA-Seq transcriptome sequencing has become an important research method to analyze the molecular mechanism of host–pathogen interactions. This technology can analyze the changes in gene expression profiles under different treatment conditions at the genome-wide level and facilitates the identification of key candidate genes associated with immune defense and metabolic regulation [32]. During fungal infection of mites, hosts initiate a series of immune responses by regulating multiple immune-related genes and signaling pathways, thereby recognizing invading pathogens and activating defense mechanisms that ultimately influence fungal infectivity and pathogenicity [33]. In studies of *Beauveria bassiana* infection in insects, transcriptomic analysis showed that detoxification enzymes, antioxidant enzymes and signal transduction-related immune genes in the host were significantly up-regulated, indicating that the host resisted pathogen infection by activating multiple immune and metabolic regulatory pathways [34]. Similarly, transcriptomic studies of *Metarhizium anisopliae*–insect interactions demonstrated that antioxidant enzyme genes, cytochrome P450 family genes and lipid metabolism-related genes were involved in host immune regulation and stress response [35].

In the present study, transcriptomic analysis showed that *I. cateniannulata* infection could up-regulate multiple immune-related genes in lysosomal, autophagy, phagosome and antigen processing-related pathways in *T. urticae*. This response may result from the host immune system recognizing conserved pathogen-associated molecular structures during fungal invasion. Previous studies have found that fungal cell wall components, such as β-1,3-glucan and chitin, can quickly trigger a series of signaling cascades in the host, thereby activating downstream immune-related pathways, enhancing antioxidant systems, lysosomal activity, phagocytosis, and autophagy-mediated cellular immune responses, ultimately suppressing or eliminating invading pathogens [36]. The immune response pattern observed in this study is generally consistent with those reported in insects and mites following fungal infection, suggesting that innate antifungal immune mechanisms are relatively conserved among arthropods.

During fungal infection, *T. urticae* exhibited differential expression of genes associated with stress resistance, immunity and lipid metabolism, with several genes showing peak expression at specific time points. Previous studies in arachnids have shown that transcriptional regulators are rapidly activated in the early stages of pathogen stimulation to regulate stress-response and immune-related pathways [37]. In this study, *TuMaf* exhibited peak expression at 12 h, suggesting its involvement in rapid stress regulation during the early stage of infection. *TuLip* and *TuCYP1* showed peak expression at 24 h, which may be due to the fact that fatty acids and lipid signaling molecules can participate in the regulation of defense responses [38]. In addition, cytochrome P450 (CYP) family genes in *T. urticae* have been closely associated with xenobiotic stress responses and detoxification and may contribute to the regulation of exogenous metabolites and oxidative stress [39,40,41,42]. The genes *TuAPOD*, *TuRbp*, *TuPLA2* and *TuCYP2* each reached peak expression at 36 h, indicating that they may mainly be involved in a sustained immune response and an oxidative stress response. The transient expression of *TuTrp* at 36 h revealed that it may be involved in host stress response and transcriptional regulation in the early stage of pathogen infection [43]. In the down-regulation stage, as infection progressed (48–72 h), the expression levels of *TuAPOD*, *TuPLA2* and *TuCYP* gradually decreased and, in some cases, fell below those of the control group. This phenomenon may be attributed to the secretion of virulence factors by entomopathogenic fungi that suppress host immune responses during infection [44]. In addition, prolonged infection may deplete host energy reserves, resulting in reduced transcriptional activity and eventual impairment or collapse of the immune system under severe infection conditions [45].

Overall, all candidate genes exhibited higher expression levels than the control group during the early and middle stages of infection (12–36 h), particularly *TuAPOD*, *TuPLA2* and *TuRbp*, which showed substantial up-regulation. These findings suggest that these genes may be the key defense factors in the antifungal response of *T. urticae*. Antioxidant-related proteins likely contribute to the elimination of reactive oxygen species (ROS) and the reduction of oxidative damage, while *the TuRbp* gene may continue to participate in the binding or transport regulation of related molecular substances during pathogen infection. In addition, PLA2 appears to mediate immune responses through lipid signaling pathways [43].

Lipid metabolism plays a key role in immune regulation in arthropods. On the one hand, fatty acids, sphingomyelins and their derivatives function as signaling molecules that activate immune pathways such as MAPK signaling, thereby promoting antimicrobial peptide synthesis and ROS production to enhance rapid host responses to pathogens [46,47]. On the other hand, lipids such as phospholipids and cholesterol provide a structural basis for the assembly of pattern-recognition receptors (PRRs) and signal complexes by regulating membrane fluidity and membrane microdomain structure [48]. This study shows that lipid metabolism-related pathways are at the core of the immune response, particularly because *PLA2* and lipid signal-related genes are significantly up-regulated, suggesting that lipid signals play an important role in amplifying immune responses [49]. Dynamic expression analysis further revealed that *TuAPOD* and *TuPLA2* exhibited an “increase-then-decrease” expression pattern after infection, reaching a peak at 36 h and showing the highest expression during the adult stage. These findings suggest that the host rapidly activates the lipid-mediated immune response in the early stage of infection, while the adult stage has a stronger immune regulation capacity [50]. Oxidative stress regulation is an important part of the host immune defense system. The cytochrome P450 (CYP) family, as a multifunctional oxidase widely present in arthropods, plays a central role in xenobiotic metabolism, detoxification and immune response [40,41,42,51,52,53]. Upon pathogenic infection, CYP450 can mediate the immune response of the host by regulating lipid metabolism, participating in the synthesis of antibacterial compounds, and scavenging excessive reactive oxygen species (ROS). Concurrently, antioxidant enzymes, such as superoxide dismutase (SOD), clear accumulated ROS to maintain redox homeostasis, thereby minimizing oxidative damage caused by intensive immune responses. Studies have shown that in arthropods, CYP450 genes are not only involved in chemical detoxification but are also closely related to immune defense and stress adaptation. For example, in Phyllotreta striolata, CYP6TH1 was significantly up-regulated under xenobiotic stress, contributing to tolerance and the maintenance of cellular homeostasis [54]. Similarly, RNAi-mediated silencing of multiple CYP450 genes can enhance the sensitivity of ticks to insecticides and disrupt their physiological equilibrium [55]. In this study, multiple antioxidant and detoxification-related genes (including the CYP450 family and SOD-related genes) were significantly up-regulated, highlighting their vital role in maintaining redox balance and reducing immune side effects. Further analysis showed that the expression levels of *TuCYP1* and *TuCYP2* reached a peak at 36 h after *I. cateniannulata* infection, reaching 5.2-fold and 3.4-fold increases relative to the control, respectively. It was speculated that *TuCYP1* and *TuCYP2* could respond to the oxidative stress induced by pathogen infection by regulating ROS levels in the middle immune response stage of the host. This expression pattern is consistent with the stress response of many arthropods under fungal infection or chemical stress.

In the immune response, transcription factors can sense pathogen infection signals and regulate the expression of downstream effector genes, which directly affect the defense ability and stress response of host cells. Trp-type transcription factors were found to be associated with antimicrobial peptide genes and stress-response networks in Helicoverpa armigera, indicating that they may play a regulatory role in the immune response [38]. In this study, the expression of *TuTrp* was significantly higher in the nymphal stage than in other developmental stages. This stage-specific profile suggests that younger individuals rely more heavily on transcriptional regulation to rapidly activate their immune system during the initial stages of pathogen invasion.

Apolipoprotein D (APOD) and phospholipase A2 (PLA2) are key functional molecules closely related to lipid metabolism and immune regulation. As a lipid-binding transport protein, insect APOD not only facilitates lipid trafficking but also functions as a pattern-recognition-related molecule involved in pathogen detection and immune activation. For example, APOD can bind β-1,3-glucan to enhance hemocyte encapsulation, phagocytosis, and AMP expression in Galleria mellonella [56,57,58]; in Spodoptera exigua, its homolog Se-ApoD3 was significantly up-regulated under immune induction and participated in the immune pre-excitation response. PLA2, a key lipid metabolism enzyme, is involved in the production of immune-associated lipid signaling molecules and regulates inflammation-like reactions. Its enzymatic activity is crucial for arthropod resistance against pathogens. Inhibition of PLA2 can significantly weaken immune responses and increase individual mortality [59,60]. In the present study, both *TuAPOD* and *TuPLA2* showed significant spatio-temporal expression dynamics, which were rapidly up-regulated and peaked at the early stage of infection (24–36 h) before gradually decreasing. This manifests a classic immune response pattern characterized by “early induction, mid-phase peak, and late-stage decline.” Furthermore, the expression of both genes was significantly more pronounced in the adult stage, suggesting that mature individuals may have stronger lipid signal mobilization and immune regulation, thus showing higher defense potential in response to pathogen infection. Therefore, APOD and PLA2 appear to act in concert to defend against pathogens by mediating lipid signal transduction and regulating immune effectors. However, the specific molecular mechanisms of these processes in mites still need further systematic investigation.

RNA interference (RNAi), which selectively suppresses gene expression by degrading target mRNA, has been widely used to characterize gene function in arthropods [61]. In this study, RNAi functional validation further demonstrated that *TuAPOD* and *TuPLA2* play vital roles in the host’s antifungal defense of *T. urticae*. After silencing *TuAPOD*, the host’s sensitivity to fungal infection was significantly increased, suggesting that it may maintain cell homeostasis by regulating lipid transport and the oxidative stress buffer system. Combined with previous studies, APOD is involved in the clearance of lipid peroxidation products and the defense against oxidative damage in a variety of organisms. Therefore, it is speculated that APOD also has a similar antioxidant protection function in *T. urticae* [49,57,58]. In contrast, *TuPLA2* silencing enhanced the lethal effect on the host more significantly, indicating that the gene plays a central role in the amplification of immune signals. Previous studies have demonstrated that *Beauveria bassiana* is an effective biological control agent against *T. urticae*, with most studies focusing on fungal pathogenicity and biocontrol efficacy [26,27]. Similarly, Bugeme et al. [62] demonstrated that *Metarhizium anisopliae* could effectively suppress *T. urticae* populations under greenhouse and field conditions. Consistent with these findings, our results showed that silencing the immune-related genes *TuAPOD* and *TuPLA2* significantly enhanced the pathogenicity of *I. cateniannulata*, resulting in higher mortality, lower escape rates, and reduced oviposition. PLA2 participates in the immune cascade by regulating the arachidonic acid pathway; hence, its loss of function may block downstream immune signal transduction, thus significantly weakening the host’s disease resistance [63]. This result is highly consistent with findings in insect systems, where PLA2 inhibition suppresses immunity [60]. In addition, combined RNAi and fungal treatment significantly reduced the number of eggs laid but had no significant effect on escape behavior, suggesting that this dual stress primarily affects physiological and energetic allocation rather than inducing behavioral avoidance. The energy balance between the immune response and reproductive activity may be an important reason for reproductive inhibition; that is, under the pressure of pathogens, the host preferentially allocates resources to survival-related pathways, thereby reducing reproductive investment [64].

Overall, this study systematically analyzed the immune response mechanism of *T. urticae* during *I. cateniannulata* infection. Using RNA-Seq technology, we comprehensively analyzed host transcriptional alterations under pathogen stress and screened a cohort of differentially expressed genes linked to immune defense and stress adaptation. Our findings reveal the coordinated activation of multi-level biological processes, including metabolic reprogramming, membrane structure remodeling, and catalytic reactions, which underscores their collective importance in the host response to pathogen challenge. Crucially, we mapped an immune response network centered on lipid metabolism regulation, oxidative stress defense, and immune signaling pathways. Furthermore, this study represents the first identification of the key genes *TuAPOD* and *TuPLA2* participating in the antifungal response of *T. urticae.* Integrative bioinformatics and RNAi functional analyses demonstrated that both genes exercise critical regulatory roles in host defense: *TuAPOD* preserves lipid homeostasis and provides antioxidant protection, whereas *TuPLA2* is indispensable for immune signal amplification. Silencing these genes significantly enhanced the virulence of *I. cateniannulata*, accelerating host mortality and suppressing fecundity. These outcomes highlight the potential of targeting key host immune genes in combination with biocontrol agents as a viable strategy for sustainable pest management. Collectively, these findings provide fundamental baseline data for clarifying mite–entomopathogenic fungus interactions, enrich our theoretical understanding of lipid metabolism genes in acarine immunity, and establish a novel theoretical foundation for developing RNAi-based green control strategies against pest mites.

## Figures and Tables

**Table 1:** Physicochemical properties.

Gene Name	Amino Acid Number	MW (kDa)	pI	Instability Index	Aliphatic Index
*TuAPOD*	194	22.05	4.74	27.11	87.42
*TuPLA2*	443	50.73	7.61	45.45	90.16

Note: Abbreviations: MW, molecular weight; kDa, kilodalton; pI, theoretical isoelectric point. The instability index was used to evaluate protein stability, with values <40 indicating stable proteins and values >40 indicating unstable proteins. The aliphatic index represents the relative volume occupied by aliphatic side chains and is considered an indicator of protein thermostability.

## Data Availability

The data that support the findings of this study are available from the corresponding author and the first author upon reasonable request.

## Supplementary Materials

The following supporting information can be downloaded at https://www.mdpi.com/article/10.3390/insects17080855/s1. Table S1: Raw data sheet for mortality, oviposition, and escape rates of *T. urticae*. Table S2: RNAi silencing efficiency of the *TuAPOD* and *TuPLA2* genes. Table S3: Spatial qRT-PCR data of each gene. Table S4: Temporal qRT-PCR data of each gene. Table S5: ref-trans-full-table.
